# Malnutrition in Devbhumi Dwarka: A Situation Analysis

**DOI:** 10.7759/cureus.27990

**Published:** 2022-08-14

**Authors:** Somen Saha, Apurvakumar Pandya, Devang Raval, Manoj S Patil, Deepak Saxena

**Affiliations:** 1 Public Health, Indian Institute of Public Health Gandhinagar, Gandhinagar, IND; 2 Adjunct Faculty, Jawaharlal Nehru Medical College, Datta Meghe Institute of Medical Sciences, Wardha, IND; 3 Epidemiology, Indian Institute of Public Health Gandhinagar, Gandhinagar, IND; 4 Psychology, Parul Institute of Public Health, Parul University, Vadodara, IND; 5 Research and Development, Jawaharlal Nehru Medical College, Datta Meghe Institute of Medical Sciences, Wardha, IND

**Keywords:** india, gujarat, devbhumi dwarka, uptake of icds and health services, nutrition status

## Abstract

Background

Malnutrition among children, adolescent girls and women is a serious public health issue in India. Malnutrition among adolescent girls is likely to carry on this vicious cycle by giving birth to low birthweight babies. Moreover, low birthweight babies who survive are likely to suffer growth retardation and illness throughout their childhood, adolescence and adulthood. Present situation analysis highlights not only the overall nutrition picture of Devbhumi Dwarka but also narrates the uptake of current nutrition and healthcare services in the Devbhumi Dwarka district.

Methods

A descriptive cross-sectional study was conducted with 3,738 study population (1,301 children, 1,185 pregnant and lactating women and 1,252 adolescent girls) across four blocks of the Devbhumi Dwarka district of Gujarat. Anthropometric assessments were carried out and the WHO Asia Pacific classification was used for the assessment of malnutrition.

Results

The prevalence of wasting, underweight and stunting was 14%, 17% and 32%, respectively, in children under two years of age. The prevalence of anaemia among pregnant women (Hb <11 g/dL) was 72.92%; 91.36% of lactating women were anaemic (Hb <12 g/dL). The prevalence of underweight (<-2 SD) among adolescent girls was 19.6%. Block-wise variation in prevalence was observed. Overall, utilization of integrated child development services and health services by these target groups remained sub-optimal.

Conclusion

The study has revealed the suboptimal nutritional status of children, pregnant and lactating women and adolescent girls of Devbhumi Dwarka, which signifies the need for urgent attention. Several areas of priority have been identified and discussed to improve the overall nutrition status in the district.

## Introduction

Malnutrition is a serious public health issue in India. According to National Family Health Survey-4 [1], the prevalence of underweight and wasted children was higher in Gujarat, which is above the national average of India. About one-third of newborns in India are born with low birth weight, and 52% of women and 74% of children are anaemic [2]. Malnutrition among adolescent girls and pregnant women is also a concern. Undernutrition among women is one of the primary causes of low birthweight babies and poor growth and is a significant contributor to infant mortality. Moreover, low birthweight babies who survive are likely to suffer growth retardation and illness throughout their childhood, adolescence and adulthood [3]. Growth-retarded adult women are likely to carry on the vicious cycle of malnutrition by giving birth to low birthweight babies [3]. It has been recognized that the long-term effects of early undernutrition and inadequate infant feeding also lead to obesity and chronic diseases, including diabetes and cardiovascular diseases [4,5].

POSHAN Abhiyaan (National Nutrition Mission) was launched in 2018 to direct the attention of the country toward the problem of malnutrition and address it in a mission mode [6]. In solidarity, Gujarat State has recently unveiled ‘Gujarat Poshan Abhiyaan 2020-22’, a state-wide two-year campaign against malnutrition. Other community-based approaches to treating undernourished and acute severe malnourishment among infants and children include providing hot meals, fortified ‘take home ration' (THR), double-fortified salt, fortified oil and fruits to Anganwadi children, pregnant women and lactating mothers. Furthermore, nutrition is one of the 11 priority health indicators of Gujarat.

Despite these efforts, progress toward reducing mortality and malnutrition has been less than needed to achieve sustainable development goals (SDGs). Most nutritional interventions covered, at best, half of the target group [7]. To achieve the priorities set by the state, responsibilities need to be shared by various stakeholders like industries and academic institutions.

Project Tushti-Towards Kuposhan Mukt Devbhumi Dwarka was launched to reduce malnutrition in Devbhumi Dwarka in 2019. Project Tushti is implemented in 249 villages with a high concentration of malnourished children, spread over four blocks of Devbhumi Dwarka. This paper reports findings from a situation analysis commissioned under this initiative. This situation analysis presents an overview of the nutrition (undernutrition) status of children under five years of age, pregnant and lactating women and adolescent girls in Devbhumi Dwarka. It briefly discusses interventions that may strengthen existing health and nutrition programmes.

## Materials and methods

A descriptive cross-sectional study was conducted in four blocks: Khambhaliya, Bhanvad, Kalyanpur and Dwarka blocks of Devbhumi Dwarka district of Gujarat. The sample size was calculated using the formula: sample size, n = [DEFF*Np(1-p)]/[(d2/Z21-α/2*(N-1)+p*(1-p)], where p is the prevalence. The calculation followed the principles used in the WHO survey and was conducted in the OpenEpi Software. The sample size for the study was 3,738, including 1,301 children, 1,252 adolescent girls and 1,185 pregnant and lactating women.

In anthropometric measurements, height and weight were included. Standard validated instruments, wooden infanto-cum-stadiometer and digital weighing scales for children were used. The nutritional status of adolescent girls was assessed using anthropometry. Height was measured using a stadiometer (nearest 0.1 cm), and weight (nearest 0.5 kg) was measured using a digital weighing machine. WHO Asia Pacific classification [8] was used for the assessment of malnutrition.

Anaemia was determined from the last Hb test record using the Mamta card. The anaemia status of the surveyed women was classified into severe, moderate, mild and any anaemia based on WHO guidelines [9]. As per National Iron Plus Initiative (NIPI) guidelines, severe anaemia level was defined as haemoglobin <7.0 g/dL for pregnant women (PW) and <8.0 g/dL among lactating women (LW). Moderate anaemia was defined as 7.0-9.9 g/dL for PW and 8.0-10.9 g/dL among LW. Mild anaemia was defined as 10.0-10.9 g/dL in PW and 11.0-11.9 g/dL in LW. Any anaemia was defined as the haemoglobin level <11.0 g/dL in PW, whereas <12.0 g/dL in LW.

Using a survey tool, detailed information on the profile of beneficiaries, practices related to general hygiene, child nutrition and feeding practices, nutrition among adolescent girls, pregnant and lactating women and access to Integrated Child Development Services (ICDS) services were gathered. The data collection tool was pilot-tested and administered by trained data collectors in regional languages.

Approval from the district authority of Devbhumi Dwarka and ethical approval from the Institutional Ethics Committee at the Indian Institute of Public Health Gandhinagar were obtained. Procedures to assure participants’ confidentiality were strictly observed.

## Results

The assessments have provided insights on the situation of nutritional status of children, adolescents and pregnant and lactating women and uptake of nutrition and healthcare services in Devbhumi Dwarka. A total of 3,738 samples were collected from 44 villages in four blocks of Devbhumi Dwarka District: Bhanvad, Khambhaliya, Kalyanpur and Dwarka. A blockwise distribution of study participants was presented in Table 1.

Demographic profile of study participants

Data represented from birth to 6-month-old children (12%), 7-24 months children (23%), adolescents (33%) and antenatal care (ANC)/postnatal care (PNC) mothers (32%). Table 2 depicts the demographic profile of the study participants. Most participants (79%) were Hindus and the rest (20%) were Muslims. Regarding caste, 63% of the sample represented other backward classes (OBCs), followed by scheduled tribes and scheduled castes (19% and 4%, respectively). Most participants (70%) belonged to joint families with an average of 4-6 family members in the family. The majority of respondents (72%) had pucca houses, whereas 80% of the respondents have availability of clean drinking water from the government and 17% have tube well or well. Out of the total, 79% of them had toilet facilities in their home and 78% of them reported using toilet facilities.

**Table 1:** Blockwise distribution of study sample (N=3,738) ANC, antenatal care; PNC, postnatal care.

Type of samples	Bhanvad	Dwarka	Kalyanpur	Khambhaliya	Total
0-6 Months child	91 (12.6)	126 (10.3)	125 (12.6)	104 (13)	446 (12)
7-24 Months child	173 (24)	303 (24.7)	234 (23.6)	145 (18)	855 (23)
Adolescent girls	243 (33.7)	450 (36.7)	328 (33)	231 (29)	1252 (33.5)
ANC and PNC mothers	214 (29.7)	346 (28.2)	306 (30.8)	319 (40)	1185 (31.7)
Total	721	1225	993	799	3738

**Table 2:** Demographic profile of study participants (N=3,738) SC, scheduled caste; ST scheduled tribe, OBC, other backward class; APL, above poverty line; BPL, below poverty line.

Sample profile	N=3,738 (%)
Religion	
Hindu	2969 (79.5)
Muslim	0749 (20)
Others	07 (0.2)
Do not want to disclose	13 (0.3)
Caste	
General	399 (10.7)
SC	132 (3.5)
ST	709 (19)
OBC	2357 (63.1)
Do not know	141 (3.8)
Socioeconomic class	
APL	2480 (66.3)
BPL	1076 (28.8)
Do not know	182 (4.9)
Family type	
Joint	2609 (69.8)
Nuclear	1129 (30.2)
Housing type	
Kutcha house	497 (13.3)
Semi-kutcha house	540 (14.4)
Pucca house	2701 (72.3)
Drinking water facility	
Tap	2990 (80)
Tube well	375 (10)
Well	262 (7)
Others	111 (3)
Sanitation facility	
Household access to toilet	2950 (78.9)
No facility	788 (21.1)

As shown in Table 3, nearly half of the participants (45%) completed primary education and 26% completed secondary education. Only 5% had higher secondary and 3% had graduation. Nearly a quarter (22%) of participants were illiterate. Two-thirds of the sample (69%) have reported to be engaged in domestic work as their occupation. About 7% of participants were labourers, 0.9% (33) were doing jobs and 0.6% (22) were entrepreneurs.

**Table 3:** Education and occupation of the study participants (N=3,738)

Education	Bhanvad	Dwarka	Kalyanpur	Khambhaliya	N (%)
Illiterate	93 (12.9)	297 (24.2)	189 (19)	224 (28)	803 (21.5)
Primary	376 (52.1)	477 (38.9)	483 (48.6)	354 (44.3)	1690 (45.2)
Secondary	202 (28)	392 (32)	241 (24.3)	138 (17.3)	973 (26)
Higher secondary	23 (3.2)	36 (2.9)	46 (4.6)	64 (8)	169 (4.5)
Graduation and above	27 (3.7)	23 (1.9)	34 (3.4)	19 (2.4)	103 (2.8)
Total	721	1225	993	799	3738
Occupation					
Domestic work	444 (61.6)	949 (77.5)	622 (62.6)	578 (72.3)	2593 (69.4)
Labourer	68 (9.4)	14 (1.1)	130 (13.1)	54 (6.8)	266 (7.1)
Service	4 (0.6)	6 (0.5)	8 (0.8)	15 (1.9)	33 (0.9)
Entrepreneur	1 (0.1)	18 (1.5)	2 (0.2)	1 (0.1)	22 (0.6)
Study	204 (28.3)	238 (19.4)	231 (23.3)	151 (18.9)	824 (22)
Total	721	1225	993	799	3738

Status of Child Nutrition and Feeding Practices

About 12.2% of babies were low birthweight (LBW) babies (Table 4).

**Table 4:** Blockwise child birthweight status (N=1,301)

Birthweight status	Bhanvad	Dwarka	Kalyanpur	Khambhaliya	Total (%)
Extremely low birthweight (<1000 g)	0 (0.0)	3 (0.7)	1 (0.3)	1 (0.4)	5 (0.4)
Very low birthweight (<1500 g)	4 (1.5)	4 (0.9)	2 (0.6)	2 (0.8)	12 (0.9)
Low birthweight (<2500 g)	23 (8.6)	53 (12.4)	47 (13.1)	36 (14.5)	159 (12.2)
Normal birthweight (2500 kg to 3500 g)	237 (88.4)	357 (83.2)	304 (84.7)	208 (83.5)	1102 (84.7)
High birthweight (>3500 g)	4 (1.5)	12 (2.8)	5 (1.4)	2 (0.8)	23 (1.8)

Variation within the block was observed: Dwarka and Kalyanpur blocks reported a higher percentage of LBW babies than others. As shown in Table 5, the prevalence of childhood wasting, underweight and stunting was 14.2%, 17.2% and 31.5, respectively.

The assessment has highlighted a higher percentage of institutional delivery (99%), but uptake of government hospitals was reported to be poor, with only 49% of children delivered in government hospitals.

**Table 5:** Nutritional status of children as per WHO’s Asia Pacific Criteria (N=1,185) SD, standard deviation.

	Bhanvad	Dwarka	Kalyanpur	Khambhaliya	N (%)
Wasting: 169 (14.2) weight for height <–2 SD of the WHO Child Growth Standards Median					
Severe	17 (6.8)	28 (7.5)	20 (5.9)	17 (7.5)	82 (6.9)
Moderate	20 (8.0)	26 (7.0)	26 (7.7)	15 (6.6)	87 (7.3)
Normal	172 (69.1)	230 (62)	220 (65.3)	161 (70.6)	783 (66)
Overweight	23 (9.2)	46 (12.4)	48 (14.2)	22 (9.6)	139 (11.7)
Obese	17 (6.8)	41 (11)	23 (6.8)	13 (5.7)	94 (8)
Total	249	371	337	228	1185
Stunting: 374 (31.5) height for age <–2 SD of the WHO Child Growth Standards Median					
Severe	36 (14.5)	66 (17.8)	42 (12.5)	27 (11.8)	171 (14.4)
Moderate	33 (13.3)	60 (16.2)	74 (22.0)	36 (15.8)	203 (17.1)
Normal	149 (59.8)	181(48.8)	180(53.4)	140(61.4)	650(54.8)
Overweight	16 (6.4)	26 (7.0)	18 (5.3)	10 (4.4)	70 (5.9)
Obese	15(6)	38(10.3)	23(6.9)	15(6.5)	91(7.7)
Total	249	371	337	228	1185
Underweight: 204 (17.2) weight for age <–2 SD of the WHO Child Growth Standards Median					
Severe	12 (4.8)	16 (4.3)	19 (5.6)	12 (5.3)	59 (5.0)
Moderate	28 (7.5)	52 (14.0)	38 (11.3)	27 (11.8)	145 (12.2)
Normal	200 (80.3)	264 (71.1)	262 (77.8)	179 (78.5)	905 (76.4)
Overweight	8 (3.2)	31 (8.4)	13 (3.9)	9 (3.9)	61 (5.1)
Obese	1 (0.4)	8 (2.2)	5 (1.5)	1 (0.4)	15 (1.3)
Total	249	371	337	228	1185

In terms of Infant and Young Children Feeding Practices (IYCF) (Table 6), 55% of mothers had started breastfeeding within an hour of delivery, while more than a quarter (about 27%) had initiated breastfeeding after more than 3 h or one day. At the time of the survey, 349 (78%) infants were exclusively on breastfeeding. Yet, 22% of children were provided liquid other than milk, which included infant formula mix (11%), plain water (5.4%), fresh animal milk (5%), fruit juice (2%), yogurt and thin porridge (0.9%), before completion of six months of a child. Exclusive breastfeeding (78.2%) was reported. Nearly 78.5% (out of 855 children) were provided complementary feeding after six months and 15% initiated complementary feeding at six months. About a quarter of mothers (25%) reported providing packed food items like fried chips, wafers, biscuits, Maggie, sugary food (5.4%) and sweet beverage to their children in the last 24 h.

**Table 6:** Infant and young child feeding practices

Early initiation of breastfeeding (N=446)						
Breastfeeding initiation	Bhanvad		Dwarka	Kalyanpur	Khambhaliya	Total (%)
Within 1 h after birth	52 (57.1)		70 (55.6)	66 (52.8)	58 (55.8)	246 (55.2)
From 1 to 3 h after birth	11 (12.1)		14 (11.10)	24 (19.2)	14 (13.5)	63 (14.1)
More than 3 h after birth	3 (3.3)		12 (9.5)	13 (10.4)	3 (2.9)	31 (7)
After one day	22 (24.2)		29 (23)	22 (17.6)	27 (26)	100 (22.4)
Did not know	3 (3.3)		1 (0.8)	0 (0)	2 (1.9)	6 (1.3)
Total	91		126	125	104	446
Feeding practices (other than breastmilk) before six months (N=206)						
Any of the liquids	19 (20.9)	31 (24.6)		24 (19.2)	23 (22.1)	97 (21.7)
Plain water	3 (3.3)	7 (5.6)		8 (6.4)	6 (5.8)	24 (5.4)
Infant formula mix	6 (6.6)	16 (12.7)		13 (10.4)	13 (12.5)	48 (10.8)
Animal milk	7 (7.7)	5 (4)		3 (2.4)	7 (6.7)	22 (4.9)
Fruit juice	2 (2.2)	3 (2.4)		2 (1.6)	0 (0)	7 (1.6)
Yogurt	0 (0)	0 (0)		2 (1.6)	2 (1.6)	4 (0.9)
Thin porridge	1 (1.1)	0 (0)		1 (0.8)	2 (1.9)	4 (0.9)
Initiation of complementary feeding (N=855)						
Child age giving any food other than breastmilk						
Before six months	9 (5.2)	16 (5.3)		21 (9)	9 (6.2)	55 (6.4)
At six months	20 (11.6)	47 (15.5)		39 (16.7)	23 (15.9)	129 (15.1)
After six months	144 (83.2)	240 (79.2)		174 (74.4)	113 (77.9)	671 (78.5)

Most mothers (80%) reported receiving pre-mixed food packets of THR, but only 47% of them used it to feed their children and more than a quarter of mothers (31%) used THR received for children for family members; some did not use for cooking (3%) and a few (0.2%) fed THR to their animals.

Status of Nutrition among Adolescent Girls

About 19.6% of adolescent girls were underweight (<-2 SD). Prevalence of underweight was higher in Dwarka, Bhanvad and Kalyanpur blocks. Table 7 depicts the block-wise distribution of BMI categories of adolescent girls.

**Table 7:** Blockwise BMI status of adolescent girls according to WHO Asia Pacific Criteria (N=1,240) BMI, body mass index.

BMI categories	Bhanvad	Dwarka	Kalyanpur	Khambhaliya	N (%)
Underweight (<18.5 kg/m^2^)	58 (4.6)	82 (6.6)	57 (4.6)	18 (1.45)	243 (19.6)
Normal weight (18.5–22.9 kg/m^2^)	162 (13.1)	305 (24.6)	234 (18.9)	153 (12.3)	854 (68.9)
Overweight (23.0–24.9 kg/m^2^)	13 (1)	41 (3.3)	32 (2.6)	24 (1.9)	110 (8.9)
Obese (≥25.0 kg/m^2^)	6 (0.5)	19 (1.5)	5 (0.4)	3 (0.2)	33 (2.6)

Adolescent girls’ knowledge about health and nutrition was assessed. Almost four-fifths of the respondents (79.6%) had not heard about iron-deficiency anaemia, nearly 70% were unaware of haemoglobin tests and 68% did not know about malnutrition. About 44% did not know the benefits of safe menstrual hygiene practices.

More than half of the participants (57%) reported receiving iron folic acid (IFA) and calcium tablets, but 10% did not consume IFA due to vomiting, black stool and the taste of the tablets. Among school-going adolescents (65.8%), about 39.5% of girls received hot cooked mid-day meals (MDM). Reasons for this gap may be other adolescent girls studying in private schools as these schools are not linked with the MDM scheme.

The data showed that the participants were involved in various activities for more than 45 min a day, including cycling (26%), walking (25%) and playing outdoor games. Around 10% of adolescent girls were having some or the other kind of addiction.

Status of Nutrition among Pregnant and Lactating Women

Of 1,185, 53% were pregnant women, whereas 47% were lactating mothers. The mean age of study participants was 25.19±3.91 for pregnant women and 25.45±4.01 for lactating women. Consumption of supplements was poor among pregnant and lactating mothers. Nearly 60% of pregnant women consumed IFA tablets, while 42% reported consuming calcium tablets (Table 8). This pattern was similar across four blocks. When compared, the consumption of IFA and calcium supplements among lactating women was low. Only 18.7% of lactating women had consumed IFA tablets and about 14.8% consumed calcium tablets. Many---81.54% of pregnant women and 75.5% of lactating women---reported receiving THR regularly. However, the use of THR was unknown.

**Table 8:** Consumption of IFA and calcium among pregnant and lactating women

Supplements consumed by pregnant women (N=623)					
	Bhanvad	Dwarka	Kalyanpur	Khambhaliya	N (%)
Iron folic acid	83 (70.9)	105 (56.1)	99 (61.9)	85 (53.5)	372 (59.7)
Calcium	59 (50.4)	72 (38.5)	71 (44.4)	62 (39.0)	264 (42.4)
Supplements consumed by lactating women (N=562)					
Iron folic acid	17 (17.5)	31 (19.5)	34 (23.3)	23 (14.4)	105 (18.7)
Calcium	13 (13.4)	20 (12.6)	31 (21.2)	19 (11.9)	83 (14.8)

## Discussion

The study has highlighted the status of malnutrition among children, pregnant and lactating women and adolescent girls in Devbhumi Dwarka. About 12.2% of babies were low birthweight; the status of wasting, stunting and underweight was 14.2%, 31.5% and 17.2%, respectively.

Initiation of breastfeeding within 1 h of delivery is considered the most crucial vaccine for child growth [8,9]. But in the present study, feeding practices such as pre-lacteal feeding, too early introduction of weaning and the use of packaged food were widely practised in the community. Similar findings were reported by Das et al. in Bihar [10] and Sabharwal in 2014 [11]. A study by Das et al. in Bihar highlighted that those practising pre-lacteal feedings were less likely to maintain exclusive breastfeeding [10]. More recently, two studies reported low exclusive breastfeeding practice found to be significantly low, marked by the introduction of animal milk and solid foods even before six months of age [12,13].

In the present study, exclusive breastfeeding (78.2%) and complementary feeding (78.5%) were reported timely, which is higher than the National Family Health Survey 4 report (54.9 and 42.7%, respectively) in the country [1]. This finding contrasts with other Indian studies in urban and rural settings [7,14-16].

The study reported that the consumption of IFA and calcium was sub-optimal among adolescent girls and pregnant and lactating women. A similar finding was reported in the study conducted in rural settings of North India by Varghese and the team [17]. Consumption of IFA and calcium tablets is linked with improved haemoglobin levels and low birthweight [18].

It was quite obvious that healthcare-seeking behaviour appeared inadequate. For example, the late onset of breastfeeding, sometimes as late as 3-4 days after delivery, deprives the infant of valuable antibodies present in colostrum, and this can hurt the child's ability to fight infections, low consumption of IFA and calcium tablets among pregnant and lactating women and adolescent girls and inappropriate use of THR. Therefore, identifying constraints to utilize nutritional and health services and effective strategies to encourage caregivers to prepare and feed appropriate food have been emphasized [19,20].

There is sufficient evidence that repeated engagement using consistent key messaging is required for any behaviour to change or to initiate new behaviour so that it becomes a practice [21]. Social Behaviour Change Communication (SBCC) has the potential to improve health and caring practices to improve maternal, newborn and child health [19-21]. Behaviour change communication interventions at the individual, community and school levels can bring behavioural change. Individual-level interventions should focus on compliance to IFA and calcium supplements, appropriate IYCF practices and uptake of health and nutrition services.

## Conclusions

The study has revealed the suboptimal nutritional status of children, pregnant and lactating women and adolescent girls of Devbhumi Dwarka. This signifies the need for urgent attention. It also reinforces a need for collaborative efforts and implementation research to enhance current interventions. Existing health and nutrition programmes may benefit from intensifying the interventions like social behaviour change communication, strengthening testing and treatment of anaemia, with a focus on pregnant women and school-going adolescents and strengthening health and nutrition services. The various behaviour change communication interventions at the individual, community and school levels can bring about the required behavioural changes. Individual-level interventions should focus on compliance to IFA and calcium supplements, appropriate IYCF practices and uptake of health and nutrition services. Morning school assemblies can be utilized for community and school-level communication to discuss ‘nutrition and anaemia’. Youth festivals organized at the school platforms can also be utilized to generate discussions and dialogue on anaemia and nutrition. Various activities can be prepared for behaviour change, including sensitization meetings for the media, school teachers and administration, faith leaders, panchayat leaders, Village Health Sanitation and Nutrition Committee (VHSNC) and so on. Mobilizing and engaging women’s Self Help Groups (SHGs) under the State Rural Livelihood Mission can be beneficial. Mobile technology can play an important role in delivering SBCC messages. Mobile application with standard behaviour change messages through culturally sensitive multimedia platforms in the local language can be an effective tool for frontline workers to ensure improvement in mother-child care practices.

Screening and testing of anaemia are important in all age groups so that appropriate treatment may be initiated as per the haemoglobin level of the individual. Strengthening the Community-based Management of Acute Malnutrition (CMAM) approach can be a crucial strategy. Monitoring of Ready-to-Use Therapeutic Food (RUTF) access and consumption followed by counselling, Mid-Upper Arm Circumference (MUAC) every 15 days and checking for medical complications once a month are essential checkpoints. Counselling mothers on IYCF should be encouraged. An education campaign for the uptake of these services in tandem with peer counselling would be the most appropriate option. Essential strengthening of the healthcare system and making it responsive to the community needs specifically to address malnutrition are the need of the hour. Operationalizing child malnutrition treatment centres and nutrition rehabilitation centres is essential. At the same time, activating effective supportive supervision and monitoring will be helpful. A monthly meeting with the District Nutrition Task Force is already mandated by the states. Efforts on education and behaviour changes, along with strengthening facility and community-based health and nutrition services, should be prioritized. Existing nutrition services need to be reviewed and re-strategized to address local needs for tackling this problem successfully.
